# The Zygomatic Buttress as an Efficient Intraoral Donor Site for Limited Maxillary Reconstructions: A Case Series and Brief Literature Review

**DOI:** 10.1155/2021/5539185

**Published:** 2021-05-03

**Authors:** Joseph Bassil, Alain Abi Sleiman, Stephanie Mrad, Ziad Noujeim

**Affiliations:** ^1^Department of Oral Surgery, Faculty of Dental Medicine, Saint Joseph University of Beirut, Lebanon; ^2^Attending Oral Surgeon, Lebanese Army Dental Department, Beirut, Lebanon; ^3^Department of Oral and Maxillofacial Surgery, Faculty of Dental Medicine, Lebanese University, Beirut, Lebanon

## Abstract

Limited maxillary defects are commonly grafted with bone blocks harvested from the symphysis or the ramus; harvesting a second surgical site in the mandible increases both operative time and patient's postoperative morbidity. To overcome these disadvantages, the zygomatic buttress (ZB) was suggested as an alternative maxillary source of autogenous bone. This intraoral donor site has a natural convex shape and can be accessed along with the recipient site through the same flap design. We report a case series describing this uncommon technique of bone harvesting from the zygomatic buttress to reconstruct limited alveolar defects in the maxilla.

## 1. Introduction

Implant placement in the correct—prosthetic and esthetic—three-dimensional position requires sufficient alveolar bone volume. However, unfavorable ridge conditions are commonly encountered, and a grafting procedure is necessary to augment alveolar bone width prior to implant placement. Several bone grafting substitutes such as allogeneic, xenogeneic, and alloplastic materials have shown successful results, yet autogenous bone remains the gold standard for alveolar bone reconstructions [1]. Two of the most common intraoral donor sites used to treat limited maxillary defects are the mandibular symphysis and ramus. This implicates the necessity of a second surgical site, more operative time, and more postoperative patient morbidity. To overcome these disadvantages, the zygomatic buttress (ZB) was suggested as a source of autogenous bone [2]. The exhaustive surgical technique describing the use of ZB graft for limited maxillary reconstructions is rarely reported in the literature.

The ZB is formed by the junction of the zygomatic process of the maxilla and the maxillary process of the zygoma. It is responsible for sustaining the forces applied to the maxilla. The quality, density, and natural convex shape of the ZB compensate for the limited bone quantity can be harvested from this region. Also, easy accessibility to the donor site and its proximity to the recipient site make it possible to perform only one flap reducing both operative time and postoperative morbidity [3, 4]. The ZB block can be fixed in direct contact to the recipient site as described in the “onlay technique” or distant from the alveolar ridge as defined in the containment and contouring “shell technique” [3, 5].

This series is aimed at describing five cases of bone harvesting from the zygomatic buttress to reconstruct limited maxillary defects.

## 2. Case Series

### 2.1. Patient Selection

Five patients who required implant placement in the maxilla and presented insufficient alveolar bone thickness, ranging from 2 to 4 mm, were enrolled in this study. Grafting procedures using bone blocks harvested from the ZB were planned to reconstruct the thin ridges before implant placement (Table 1). All patients signed an informed consent agreement before surgery, and all work was conducted in accordance with the Declaration of Helsinki. Because the study was retrospective, it was granted a written exemption by the ethical committee of the Saint Joseph University of Beirut IRB.

Inclusion criteria were as follows: partial or complete maxillary edentulism, horizontal ridge deficiency, and noncontributory medical history.

Exclusion criteria were as follows: smoking, pregnancy, breast-feeding, systemic diseases, and poor oral hygiene.

### 2.2. Surgical Technique

**Figure 1:**
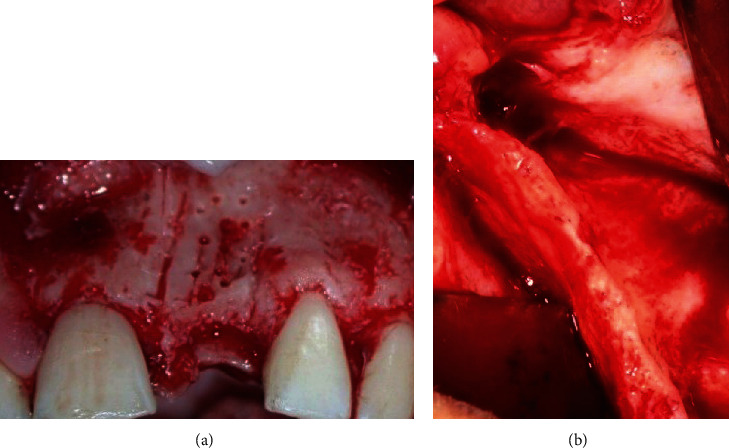
Preoperative view of the deficient maxillary recipient sites: (a) case no. 1: partially edentulous anterior site; (b) case no. 2: fully edentulous posterior site.

After local anesthesia infiltrations, a linear crestal incision was made along the edentulous ridge and was continued by intrasulcular incisions around the adjacent teeth and two mesial and distal vertical release incisions. The distal vertical release was continued into a horizontal incision in the maxillary vestibule, 3 to 5 mm above the mucogingival junction. Wide trapezoidal full-thickness flaps were raised to expose the thin maxillary ridges (ranging from 2 to 4 mm) and the ZB (Figure 1).

**Figure 2:**
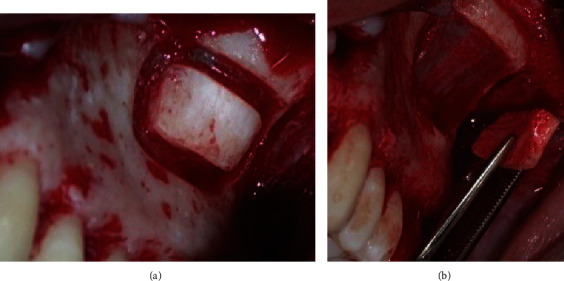
View of the donor site—lower tail of the ZB—in case no. 1: (a) bone cut with the piezoelectric surgical device; (b) ZB bone block harvest.

Bone blocks of approximately 1.5 cm^2^ were harvested from the ipsilateral ZB tail using Piezosurgery (Mectron®; Carasco-GE, Loreto, Italy) (Figure 2).

**Figure 3:**
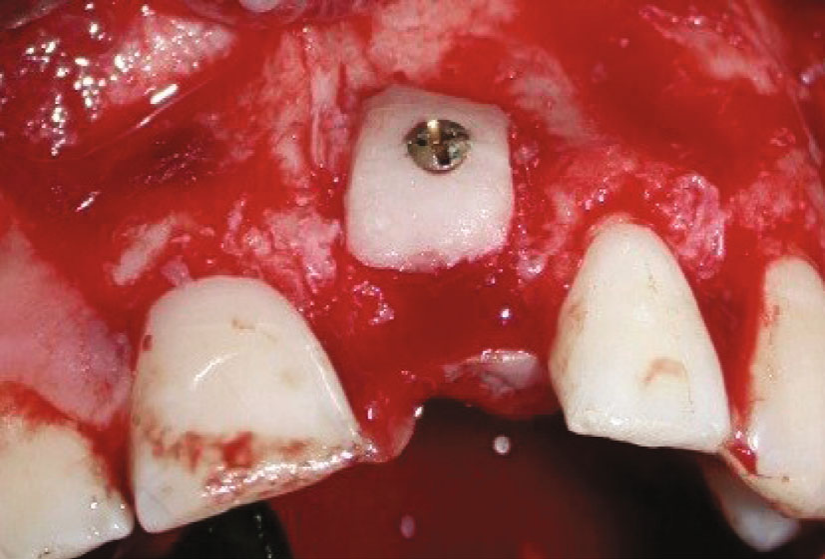
Onlay grafting procedure in case no. 1: ZB graft placed in direct contact with the recipient site and stabilized with a miniscrew.

In 2 cases, the “Onlay graft technique” was applied (Table 1): thick cortico-cancellous bone blocks harvested from the ZB were fixed, without further shaping, in direct contact with the concave alveolar defects using two titanium osteosynthesis miniscrews (smartDrive®; KLS Martin Group, Tuttlingen, Germany) (Figure 3). Autogenous bone chips were collected from the outer cortex of the same area using a bone scraper (Safescraper® Twist; CGM SpA, Divisione Medicale META, Reggio Emilia, Italy) and were mixed with anorganic bovine bone (Bio-Oss®, Geistlich Pharma AG, Wolhusen, Switzerland) particles in equal quantities to cover the outer surface of the blocks.

**Figure 4:**
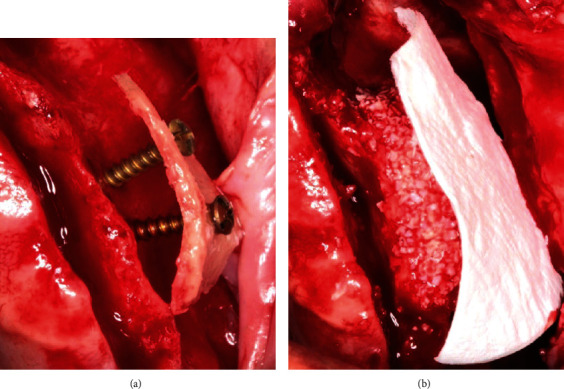
Shell grafting procedure in case no. 2: (a) ZB graft placed at a distance from the recipient site and stabilized with two miniscrews; (b) gap filled with 50 : 50 mixture (autogenous+xenogeneic) bone particles and covered with a large collagen membrane.

In the 3 other cases, the “Shell technique” was applied (Table 1): thin cortical bone blocks were harvested from the ZB and fixed at a distance from the recipient sites with titanium screws (Figure 4). In one of these cases, a simultaneous sinus floor elevation was performed to ensure sufficient bone height for later implant placement. In another advanced fully edentulous case, bone blocks harvested from the bilateral ZB were fixed to the right and left recipient sites using the shell/tenting technique. The 50 : 50 ratio mixture of bone particles (autogenous+xenogeneic) was used to fill the gaps between the bone blocks and the alveolar ridges.

In all cases, the grafted sector was covered with a large collagen membrane (Bio-Gide®, Geistlich Pharma AG, Wolhusen, Switzerland) to optimize bone remodeling and growth (Figure 4). Perforation of the Schneiderian membrane did not occur in any case. Periosteal releasing incisions of the full thickness flap allowed tension-free wound closure.

**Figure 5:**
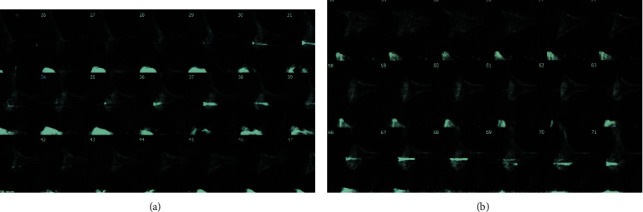
Sagittal view of CBCT images in case no. 2: (a, b) grafted areas 6 months postoperatively.

After six months of uneventful healing, cone beam computed tomography (CBCT) scans were taken to evaluate the amount of bone gain (Figure 5).

**Figure 6:**
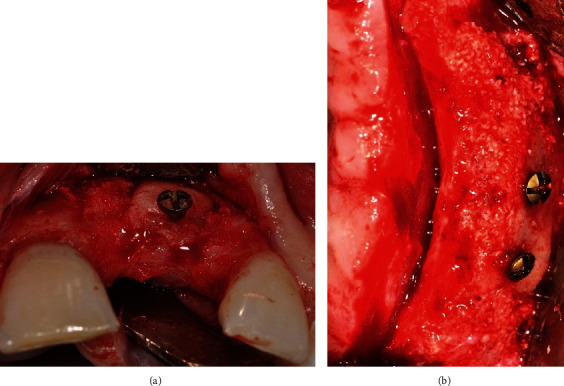
View of the grafted sites 6 months postoperatively: (a) case no. 1; (b) case no. 2.

The grafted areas were surgically reopened for conventional implant placement. After local anesthesia, a small trapezoidal full-thickness flap was raised, and the miniscrews were removed. The grafted area showed a large amount of regenerated alveolar bone with preserved convex shape in all cases (Figure 6).

**Figure 7:**
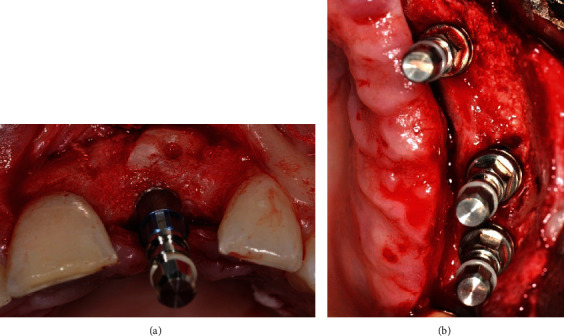
Implant placement in the grafted sites: (a) case no. 1; (b) case no. 2.

Regular-diameter (4.1 mm) Straumann® dental implants (Institut Straumann® AG, Basel, Switzerland) were placed in 4 cases according to the ideal prosthetic axis. In one case, a narrow-diameter (3.3 mm) Straumann® dental implant (Institut Straumann® AG, Basel, Switzerland) was placed for prosthetic and esthetic considerations even though the placement of a wider implant was possible (Figure 7). Implants were restored two months later with cement-retained porcelain fused to metal (PFM) crowns after abutments' tightening.

## 3. Discussion

Bone grafting procedures are commonly needed to ensure adequate bone volume before implant placement. Among several bone substitutes, autogenous bone remains the gold standard for alveolar bone reconstructions. The retromolar and symphysis intraoral donor sites are indicated in cases of large reconstructions in the mandible, whereas the ZB is described as a relatively novel intraoral donor site for small and limited bone augmentations in the maxilla [1, 6]. Gellrich et al. stated that 1.5 to 2 cm^2^ of bone can be harvested from this site without compromising midfacial strength. This quantity of harvested bone is sufficient to reconstruct limited to medium alveolar defects (sufficient for 1 or 2 implants) [3]. Kainulainen et al. also measured the volume of 40 blocks harvested from the ZB of 20 cadavers of an elderly population. Bone was either compressed into a syringe or placed in a water displacement tube to quantify its volume. An average of 0.59 mL bone was calculated with the syringe and 0.53 mL with the water displacement. It is important to note that grafts in this study were harvested from cadavers of an elderly population with moderately atrophic facial bone [4].

The ZB technique presents several advantages. A study on ten cases reported a mean horizontal bone gain of 1.82 ± 0.16 mm, 4 months after grafting with bone harvested from the ZB. These grafted sites showed similar bone density values to those of the native alveolar bone [7]. This ZB also offers bone of good quality and correct convex morphology for maxillary defects. In several studies, bone harvested from the ZB was grafted without further shaping and successfully restored a pronounced ridge contour [5]. In this series, the form-giving zygomatic graft was used in two cases of limited maxillary defects in the anterior region and ideally restored the original convex alveolar contour without any additional need for bone or soft tissue graft. The ZB method shows minimal postoperative morbidity at the donor site for no muscles need to be detached to access this site [3]. Patients grafted from the ZB were reported to encounter few postoperative difficulties, such as pain and edema, while patients grafted from the retromolar area were reported to experience more complications including transient postoperative paresthesia of the mandibular and lingual nerves [1, 5, 8]. Also, access to the ZB area is relatively simple and provides excellent visibility compared to other intraoral donor sites such as the retromolar region. Besides, the proximity between donor and recipient sites helps to simplify the grafting steps and reduce the surgical time [4].

The main limiting factors of this bone harvesting site are its close relationship to the infraorbital foramen and to the Schneiderian sinus membrane. The use of piezo surgical devices instead of rotary instruments can reduce the risk of membrane perforation. Kainulainen et al. found 33% perforated membranes among 40 surgical sites when using round burrs, while Gellrich et al. found a total of 28% perforations among 273 sites when using piezosurgery [4, 5]. However, membrane perforation did not influence the overall success of this technique. In the present series, bone harvesting was performed using a piezoelectric device to minimize the complication risk. Membrane perforation did not occur in any case.

Another key anatomic point to bear in mind when harvesting bone from the ZB is the infraorbital foramen from which the infraorbital nerve (ION) and artery (IOA) exit from the skull. Gellrich et al. propose direct visualization of the infraorbital zone to avoid any nerve injury [3]. However, this complication is rarely encountered. Sakkas et al. reported 1.7% of transient infraorbital nerve paresthesia at the time of suture removal (2 cases among 113), and all cases showed complete recovery at the time of implant placement [8]. In another retrospective study on 273 patients, no case of nerve damage was found [5]. This is in accordance with our series where no case of infraorbital nerve damage was encountered. In fact, flap retractors were positioned away from the surgical zone to avoid stretching, compressing, or lacerating the ION and IOA. Thus, the risk of bleeding or paresthesia of the ipsilateral upper lip and/or lower eyelid was minimized. The same precautions were taken when performing vertical releasing incisions that may intersect with the IOA and ION. Therefore, it is advisable not to extend the vertical incisions and to use Metzenbaum dissecting scissors for periosteum release before tension-free wound closure.

Complications related to this procedure may include infection, pus, and wound dehiscence and incision-line opening, swelling, and graft mobilization. In a study reporting complication rates of this technique, Sakkas et al. found more postoperative complications at recipient sites (17.6%) than donor sites (3.5%). Also, a significantly higher incidence of postoperative complications was found among smokers [8]. In the current report, all patients were nonsmokers and no complications were recorded confirming the previous findings. A systematic review evaluating the complications of bone harvesting from mandibular sites found high complication rates at the symphysis and the ramus [9], whereas several studies describing bone harvesting from the ZB showed very low complication and failure rates that did not significantly influence the success of this method [4, 5, 8]. Hence, the ZB appears to be a safe intraoral harvesting site.

## 4. Conclusion

The zygomatic buttress is a potential intraoral donor site of autogenous bone in cases of small or limited maxillary defects. The size and convex shape of these blocks are ideal to reconstruct the original contour of anterior maxillary defects. Proximity to the Schneiderian membrane and to the infraorbital nerve and artery should be taken into consideration during this surgical procedure. Still, this safe method presents very low complication and failure rates. The ZB donor site is close to the recipient site and can be accessed through the same flap design. This simple access will reduce the operating time and postoperative discomfort and morbidity.

## Figures and Tables

**Table 1:** Description of each case.

Case no.	Sex	Age	Graft side(s)	Grafting technique	Implant position(s)	Implant diameter (in mm)
1	Male	21	Left	Onlay graft technique: cortico-cancellous ZB bone block	21	3.3 mm (for prosthetic and esthetic considerations only)
2	Male	49	Right	Shell technique+sinus elevation	14, 15, 16	4.1 mm
3	Male	41	Left	Shell technique: cortical ZB bone block	25, 26, 27	4.1 mm
4	Female	55	Left+right	Bilateral shell technique	17, 16, 13, 23, 25, 26	4.1 mm
5	Female	49	Right	Onlay graft technique: cortico-cancellous ZB bone block	14, 15	4.1 mm
